# Efficacy and safety of combination of curcuminoid complex and diclofenac versus diclofenac in knee osteoarthritis: A randomized trial

**DOI:** 10.1097/MD.0000000000019723

**Published:** 2020-04-17

**Authors:** Dhaneshwar Shep, Chitra Khanwelkar, Prakashchandra Gade, Satyanand Karad

**Affiliations:** a Department of Pharmacology, Krishna Institute of Medical Sciences, Satara; b Department of Pharmacology, Dr. Vithalrao Vikhe Patil Foundation's Medical College & Hospital, Ahmednagar; c Department of Orthopedics, City Care Accident Hospital, Parli Vaijnath, Beed, Maharashtra, India.

**Keywords:** anti-ulcer effect, curcuminoids, diclofenac, knee osteoarthritis, pain

## Abstract

**Background:**

To compare the efficacy and safety of combination of curcuminoid complex and diclofenac vs diclofenac alone in the treatment of knee osteoarthritis (OA).

**Methods:**

In this randomized trial, 140 patients of knee OA received either curcuminoid complex 500 mg (BCM-95) with diclofenac 50 mg 2 times daily or diclofenac 50 mg alone 2 times daily for 28 days. Patients were assessed at baseline, day 14 and day 28. Primary efficacy measures were Knee injury and OA outcome score (KOOS) subscale at day 14 and day 28. Anti-ulcer effect and patient-physician's global assessment of therapy at day 28 were included as secondary endpoints. Safety after treatment was evaluated by recording adverse events and laboratory investigations.

**Results:**

Both treatment groups showed improvement in primary endpoints at each evaluation visit. Patients receiving curcuminoid complex plus diclofenac showed significantly superior improvement in KOOS subscales, viz. pain and quality of life at each study visit (*P* < .001) when compared to diclofenac. Less number of patients required rescue analgesics in curcuminoid complex plus diclofenac group (3%) compared to diclofenac group (17%). The number of patients who required histamine 2 (H2) blockers was significantly less in curcuminoid complex plus diclofenac group compared to diclofenac group (6% vs 28%, respectively; *P* < .001). Adverse effects were significantly less in curcuminoid complex plus diclofenac group (13% vs 38% in diclofenac group; *P* < .001). Patient's and physician's global assessment of therapy favored curcuminoid complex plus diclofenac than diclofenac.

**Conclusion:**

Combination of curcuminoid complex and diclofenac showed a greater improvement in pain and functional capacity with better tolerability and could be a better alternative treatment option in symptomatic management of knee OA.

**Trial Registration:** ISRCTN, ISRCTN10074826

## Abstract 2

Supplemental Digital Content is available in the text

## 1 Introduction

Osteoarthritis (OA) is the most common form of arthritis and a leading cause of disability worldwide, affecting approximately 10% of the population worldwide.^[1]^ While OA can occur at almost any joint, OA of the knee is the most common type.^[2]^ World Health Organization (WHO) report on the global burden of disease indicates that knee OA is likely to become the fourth most important global cause of disability in women and the eighth most important in men.^[3]^ The estimated prevalence of knee OA in populations above the age of 65 is 30%.^[4]^

In OA of knee, pain is the key symptom that drives individuals to seek medical attention, and contributes to functional limitations and reduced quality of life.^[5–8]^ Current recommendations for managing OA focus on relieving pain, improving physical function and to slow the progress of the underlying disease as important goals of therapy. The first line pharmacologic therapy for OA of knee including is nonsteroidal anti-inflammatory drugs (non-steroidal anti-inflammatory drugs [NSAIDs]) which provides effective relief of symptoms in most patients of knee OA.^[9,10]^ Traditional NSAIDs can reduce pain and inflammation associated with OA of knee by inhibiting cyclooxygenase-1 and cyclooxygenase-2 (COX-1 and COX-2).^[10]^ However, long-term use of NSAIDs has been found to be associated with enhanced risk for gastrointestinal (GI) bleeding, hypertension, congestive heart failure and renal insufficiency, among other adverse effects.^[11]^ Chronic administrations of NSAIDs cause gastroduodenal mucosal erosions in approximately 35% to 60% of patients, gastric or duodenal ulceration in 10% to 25% of patients.^[12]^ These events are consequence of non-selective mechanism of action of traditional NSAIDs.^[10]^ COX-2 selective inhibitors were introduced in 1999, providing analgesia equivalent to older NSAIDs with markedly lower rates of GI ulcers and related complications. A meta-analysis of all randomized, controlled trials of COX-2 selective inhibitors confirmed their increased cardiovascular risk compared with placebo. The risk was similar to the increased cardiovascular risk seen with traditional NSAIDs. Because of the high incidence of adverse events (AE) associated with both non-selective and COX-2 selective NSAID therapy, effective and safer alternative treatments for OA are required and sought.^[11]^

Turmeric (*Curcuma longa*) has been used for centuries in traditional Chinese and Ayurvedic medicine and known for its wide spectrum of pharmacological and biological activities.^[13]^ Curcuminiods are the major phytoconstituents present in the rhizomes of turmeric containing three major components (curcumin, demethoxycurcumin and bisdemethoxycurcumin). Several clinical trials have confirmed that curcumin has anti-inflammatory and analgesic properties.^[14]^ A clinical trial conducted by Kuptniratsaikul et al, has shown that the efficacy of curcumin (2000 mg/day) is similar to that of ibuprofen (800 mg/day) for the treatment of knee OA.^[15]^ Further preclinical evidence have confirmed that curcumin is gastroprotective agent and acts as a potent antiulcer compound, protecting against gastric mucosal injury.^[16,17]^ Ishita Chattopadhyay et al., have shown that curcumin acts as a potent antiulcer compound to protect indomethacin (NSAID)-induced gastric ulcer.^[18]^ It inhibits increased acid secretion to prevent ulcer aggravation. Moreover, several lines of clinical evidences confirmed that curcumin is safe for human use.^[19,20]^

However, poor oral absorption and rapid metabolism of curcumin severely curtails its bioavailability limiting its therapeutic efficacy which is a major concern. One of the approaches to increase the bioavailability of curcumin is to combine curcuminoids with essential oil of turmeric. Studies on curcuminoids combined with essential oil of turmeric shown that presence of curcumin in blood plasma was seven times higher than free curcumin and also retained in significant levels even at 8 hours post administration and found to be non-toxic and safe.^[21–23]^ Curcuminoids and essential oil of turmeric combination exhibit immense biological properties like anti-inflammatory, anti-depressant activity and radio-protective activity.^[24–26]^

Diclofenac is commonly prescribed NSAID and curcumin have been extensively used in traditional medicine in India, particularly as an anti-inflammatory agent.^[27]^ Combination of drugs with different mechanisms of action or pharmacokinetics may be more effective and less toxic than each of the monotherapeutic regimens alone.^[14]^ NSAIDs are often co-administered with proton-pump inhibitors or histamine 2 blockers (H2) blockers to reduce NSAID induced GI AE. Evidence on the clinical effectiveness of curcuminoid complex and diclofenac in patients with knee OA is lacking.

The objective of this study was to evaluate the efficacy and safety of combination of curcuminoid complex and diclofenac vs diclofenac alone in patient with OA of knee. We also investigated the anti-ulcer effect of curcuminoid complex on the basis of reduction in use of H2 blockers among Indian patients with OA of knee.

## 2 Methods

### 2.1 Ethics

The study was conducted in accordance with ethical guidelines outlined in Helsinki Declaration of 1964 as per revised version, thus ensuring greater protection to the patient. Institutional ethics committee approval was obtained from Krishna Institute of Medical Sciences, Karad, Maharashtra, India (Reference No: kimsu/PhD/11/2010) before initiating the study. Prior to any study-related screening procedures, written informed consent was obtained from each patient before enrolling in the study. The study was registered with the ISRCTN registry (ISRCTN10074826).

### 2.2 Study design and participants

In this prospective, randomized, open-label parallel group study, the patients of either gender (aged 38–65 years) suffering from symptomatic OA of knee for at least 3 months with no joint deformities and requiring treatment with anti- inflammatory drugs were screened for eligibility after taking written informed consent. Patient meeting the American College of Rheumatology criteria for OA of the knee (confirmed by X-ray) and having moderate pain (Visual analogue scale score 4 or greater) in disease joint were included in the study. Patients taking analgesics were given a washout period of at least 3 to 7 days (or longer depending on the pharmacokinetic of drug) before starting the study drug.

Patient who received corticosteroid injection of any drug within last 4 weeks; had history of active peptic ulcer, gastric ulceration, stomach pain or GI bleeding or bleeding disorders; had secondary OA due to syphilis, metabolic bone disorder, acute trauma; patients who required prescription anticoagulants, hydantoin, lithium, steroids, methotrexate and colchicines or concurrent pain relieving medication such as tranquilizers, hypnotics, excessive alcohol or any other drug affecting the evaluation of analgesic action, or patient having known hypersensitivity to diclofenac sodium and turmeric were excluded from the study. The patients with medical history of significant impairment of hepatic or renal functions, cardiac insufficiency, and bronchitis were also excluded. Pregnant and lactating women and women of child bearing age not practicing or not willing to use contraceptive were not included.

### 2.3 Interventions and dosage

Patients fulfilling the eligibility criteria were enrolled and randomized to receive either diclofenac 50 mg tablet (manufactured by Lupin Pharmaceuticals, Mumbai) twice daily or curcuminoid complex 500 mg plus diclofenac 50 mg (individual capsule and tablet was administered simultaneously, twice daily for 28 days (4 weeks).

Curcuminoid complex was provided as 500 mg zero size hard gelatin capsule (BCM-95 from Arjuna Natural Pvt. Ltd, India). Each capsule contains curcuminoids and essential oil complex (Curcumin, demethoxycurcumin, bisdemethoxycurcumin and Volatile oils from Turmeric Rhizome) total not less than 95%, curcuminoids not less than 88% and curcumin not less than 68%.

Patients were provided Paracetamol 500 mg tab (manufactured by GlaxoSmithKline Pharmaceuticals Limited) and Ranitidine 150 mg tab (manufactured by J B Chemicals & Pharma Ltd, Mumbai, India) as rescue medication as and when required.

Simple randomization sequence was generated by an independent statistician using GraphPad software with equal distribution (allocation ratio 1:1). Allocation was concealed using sequentially numbered identical boxes. Pharmacist designated by the investigator dispensed the investigational products to randomized and eligible patients.

### 2.4 Assessments

Patients were evaluated for efficacy at week 2 (day 14) and week 4 (day 28) after start of the study treatment from baseline. Primary endpoint was assessment of knee injury and osteoarthritis outcome score (KOOS) subscale at each evaluation visits. KOOS is a questionnaire designed to assess patient-relevant outcomes following knee injury. The KOOS's 5 subscales are scored separately: Pain (9 items); Symptoms (7 items); Function in daily living (17 items); Function in Sport and Recreation (5 items) and Quality of Life (4 items). A Likert scale is used and all items have 5 possible answer options scored from 0 (None) to 4 (Extreme) and each of the five subscale scores is calculated as the sum of the items included. Scores are transformed to a 0 to 100 scale, with zero representing extreme knee problems and 100 representing no knee problems. Secondary endpoints were to determine patient's global assessment for overall symptom relief and physician's global evaluation (Excellent / Good / Fair / Poor) of treatment and anti-ulcer effect. Anti-ulcer effect was assessed by recording the number of patients consumed H2 Blocker tablets during study period. Number of patients who consumed paracetamol as a rescue medication during study period was recorded at day 14 and day 28. For safety assessment, AE reported/observed during study period were recorded at each study visit. Further, laboratory-based safety assessment was done on the basis of change in hemogram (hemoglobin, RBC Count, WBC count, Differential WBC count, ESR), liver function test and renal function test at Day 28 from baseline.

### 2.5 Statistical analysis

Sample size calculation was performed using software PS Power and Sample Size Calculations (version no. 3). Based on a power of 80% and a Type I error rate of alpha = 0.05 (2-tailed), a sample size of 65 participants per group was required to detect an estimated difference of 1.24 in the mean pain scores between the treatment arms with standard deviation (SD) of 2.5. Assuming dropout rate of 5%, a total sample size of 69 participants per treatment group was considered sufficient in this study. All statistical analyses were performed on intention-to-treat basis with last observation carried forward method. Unpaired *t* test or Mann Whitney test was used to compare the data between groups and paired *t* test or Wilcoxon signed rank test were used for within group analysis of the continuous data based on distribution of data and Chi-square test or Fishers exact test was used to compare the categorical data of study groups. A comparison of 2 treatments (curcuminoid complex plus diclofenac and diclofenac alone) with the perfect analgesic was also done and the correlation coefficient was determined. *P* value of less than .05 was considered as statistically significant. All statistical analyses were performed using software, SPSS version 24.

## 3 Results

### 3.1 Patient disposition and characteristics

One hundred sixty-one patients were screened and 150 patients were enrolled in the study. A total of 140 patients (curcuminoid complex plus diclofenac: 71; diclofenac: 69) completed the study and were subjected to statistical analysis. Both treatment groups were comparable in terms of demographic characteristics, that is, age, weight, height, and gender. Clinical assessment of pain on Visual Analog scale and KOOS subscale at the start of the trial (baseline) was similar between both treatment groups. Overall, demography and baseline characteristics between both the treatment group was similar before start of study treatment (Table 1).

**Table 1:**
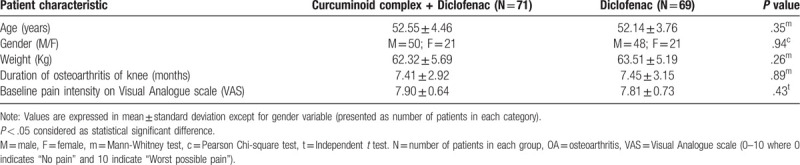
Demography and baseline characteristics in patients with OA of knee.

### 3.2 Efficacy results

Patients receiving curcuminoid complex plus diclofenac reported significantly greater improvement in KOOS score of subscales, viz. pain, symptoms and quality of life than those receiving diclofenac at the end of study (*P* < .001), except for daily living (*P* = .03) and sports/recreation activities (*P* = .36). Improvement in daily living and sports/recreational activities was numerically higher in patients treated with curcuminoid complex plus diclofenac than those receiving diclofenac (Table 2).

**Table 2:**
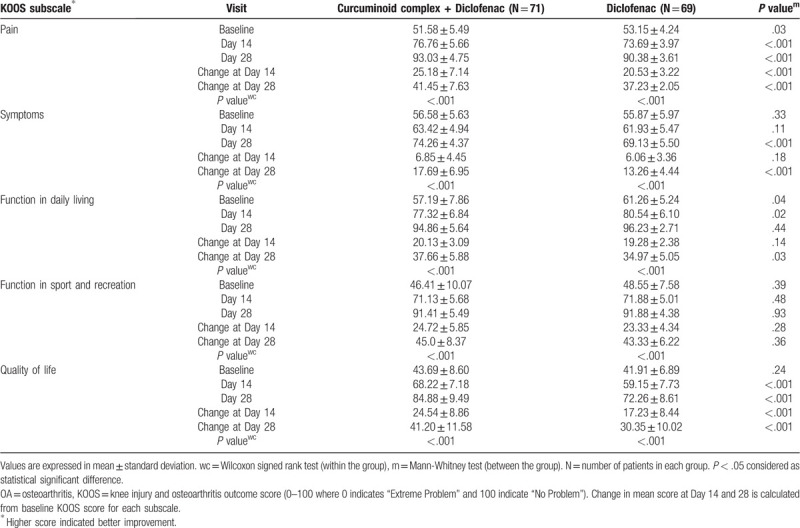
Assessment of KOOS subscale in patients with OA of knee.

**Figure 1:**
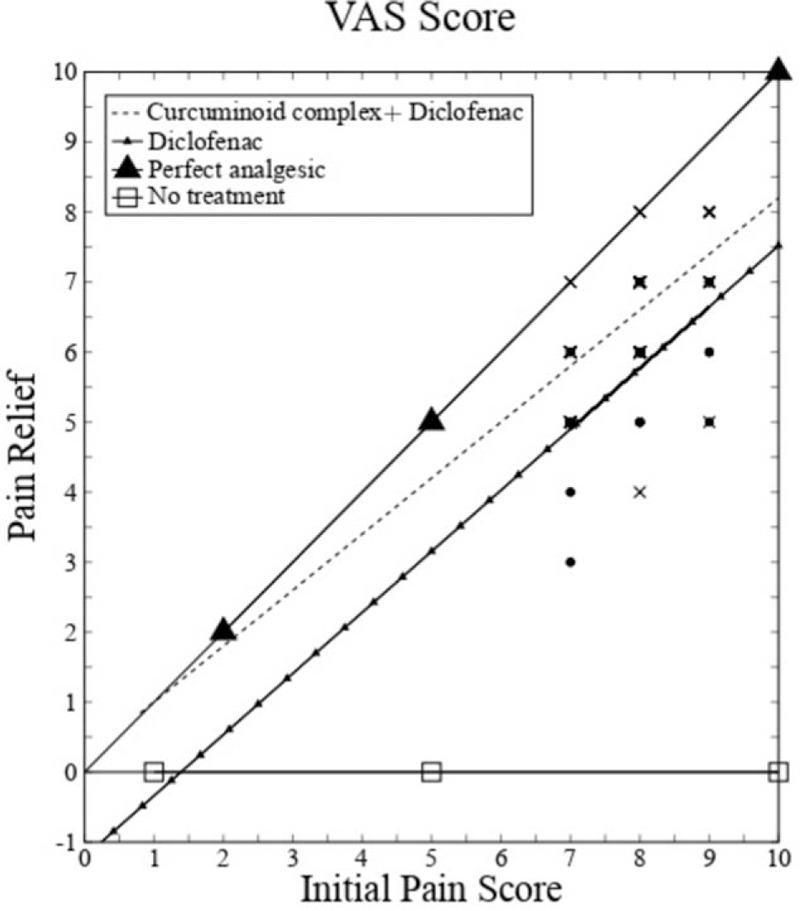
Pain relief for curcuminoid complex + Diclofenac and Diclofenac (correlation coefficient – curcuminoid complex + Diclofenac: 0.57; Diclofenac: 0.73).

In our study, pain relief for curcuminoid complex plus diclofenac and diclofenac alone are well below the “perfect analgesic” (slope = 1) and above “no treatment” (slope = 0) graph and the contribution of slope change due to the initial pain score is practically nil and statistically not significant (*P* = .43) (Fig. 1). Patients treated with curcuminoid complex plus diclofenac experienced significantly greater reduction (*P* < .001) in pain intensity at day 14 (3.73 ± 0.83) and at day 28 (4.58 ± 0.60) compared to those treated with diclofenac alone at day 14 (1.38 ± 0.74) and day 28 (2.20 ± 0.61).

The number of patients who required H2 blockers was significantly less in curcuminoid complex plus diclofenac group compared to diclofenac group (6% vs 28%, respectively; *P* < .001), this indicates anti-ulcer effect of curcuminoid complex. The need for rescue medication (paracetamol) was significantly lesser (*P* < .005) in curcuminoid complex plus diclofenac (2 patients; 3%) compared to diclofenac group (12 patients; 17%) (Table 3).

**Table 3:**
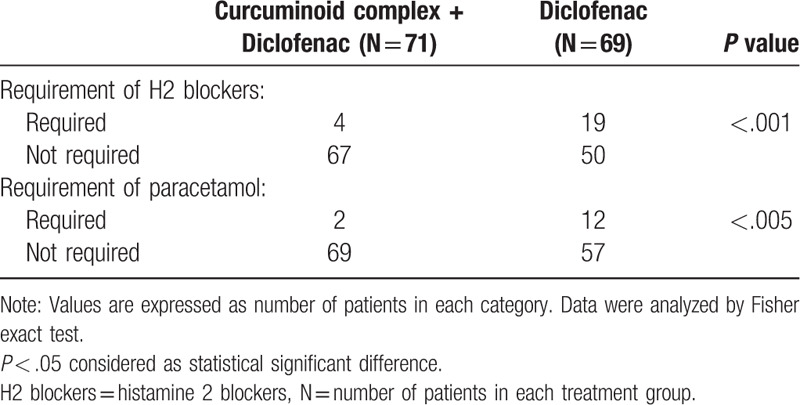
Usage of rescue medicines.

Global assessments of treatment by patient and physician based on overall efficacy and safety was favorable towards curcuminoid complex plus diclofenac compared to diclofenac alone. Greater proportion of patients rated curcuminoid complex plus diclofenac (30%) as excellent than diclofenac (17%) (Table 4).

**Table 4:**
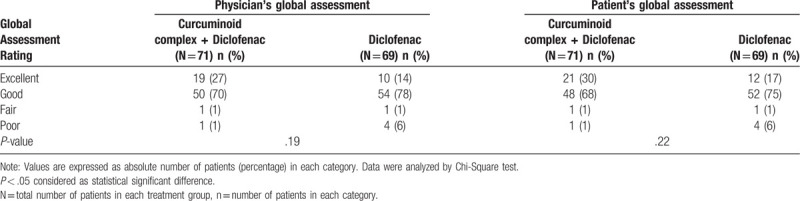
Global assessment by physicians and patients after study drug treatment.

### 3.3 Safety variables

Overall, 13% of patients receiving curcuminoid complex plus diclofenac and 38% of patients receiving diclofenac reported adverse effects, the difference was statistically significant (*P* < .001). All reported adverse effects were mild and transient. The most common adverse effects were nausea, diarrhea and abdominal pain/acidity, however the incidence of each adverse effect was significantly lesser in curcuminoid complex plus diclofenac compared to diclofenac group. Relative risk is statistically and clinically significant in abdominal pain and flatulence though only clinical significance was observed in all other AE except in nausea and diarrhea which were neither clinically nor statistically significant (Table 5). There was no significant change in blood reports with respect to complete blood count, kidney function and liver function before and after use of study medications (Table 6).

**Table 5:**
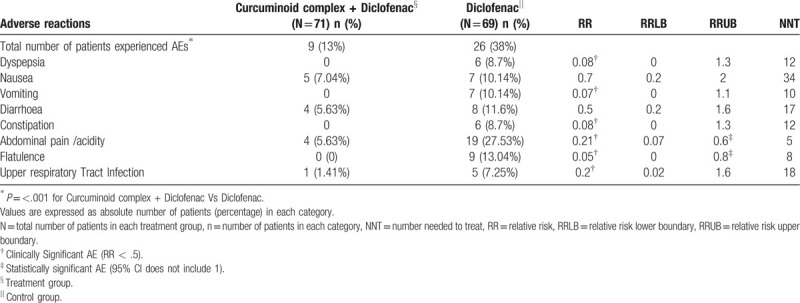
Effect of combination of curcuminoid complex with diclofenac on adverse events.

**Table 6:**
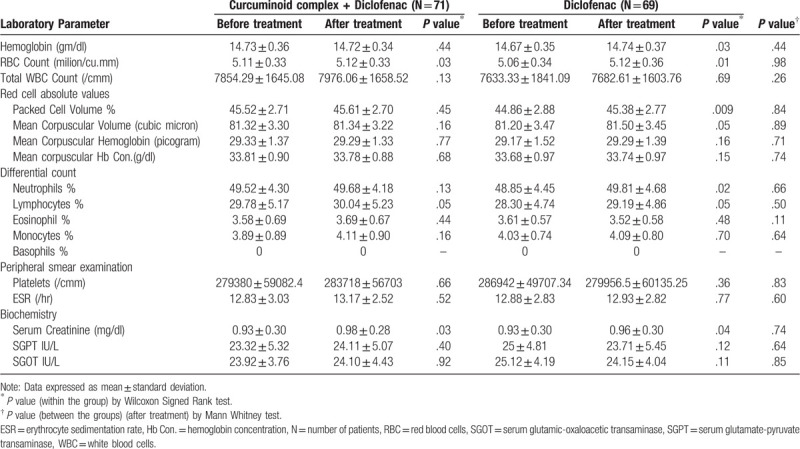
Laboratory-based evaluations of safety.

## 4 Discussion

Currently available medication regimens for treatment of OA of knee include non-opioid analgesics such as acetaminophen and NSAIDs, including COX-2 inhibitors. However, long-term use of NSAIDs has been found to be associated with enhanced risk for GI bleeding, hypertension, congestive heart failure, and renal insufficiency.^[11]^ There is need of effective and safer alternative treatments for patients with OA of knee.

This study demonstrated that curcuminoid complex plus diclofenac administered twice daily for 28 days in patients with OA knee was superior to diclofenac administered twice daily for KOOS subscale score for pain at each evaluation visits indicating better pain relief with combination of curcuminoid complex and diclofenac. Apart from pain relief, treatments with curcuminoid complex plus diclofenac also resulted in significant improvement in functional mobility and overall quality of life in patients of OA knee.

Many researchers have described the dark side of curcumin. The major drawback was its poor pharmacokinetic and pharmacodynamic properties.^[28,29]^ Meta-analysis of data from clinical trials with curcumin supplementation reported that with respect to treatment duration, the reduction in pain severity did not reach statistical significance. The author confirmed by the results of subgroup analysis that analgesic effect of curcuminoids may be greater with bioavailability optimized preparations.^[30]^ Study conducted with combination of curcumin and diclofenac showed improvement in pain and KOOS score but did not reveal any statistically significant difference and the author opinioned that it may be due to the inadequate dose of curcumin used.^[31]^ The inadequate results may be due to the poor bioavailability of curcumin which is a major hindrance to its therapeutic efficacy. Beneficial results obtained with combination therapy of diclofenac and curcuminoid complex in our study is possibly due to the presence of curcuminoids and essential oil of turmeric with turmerones which enhanced the bioavailability of curcumin.

Turmerones acts by inhibiting p-glycoprotein, thus increasing the permeability of curcumin.^[32]^ The combination of curcuminoids with turmerones was reported as a powerful tool in prevention of inflammation associated colon carcinogenesis than curcuminoids or turmerones alone, exhibiting its synergic or additive effect.^[33]^ Many published papers confirm the superiority of curcuminoids with essential oil of turmeric with turmerones. Combination of curcuminoid essential oil of turmeric showed superior protection from dextran sodium sulfate (DSS)-induced colitis than curcumin alone, highlighting its synergic and anti-inflammatory potential.^[34]^ Study done by dietary supplementation of curcuminoid essential oil complex reduced the inflammatory state in obese cats by showing beneficial effect on inflammatory markers expressed by peripheral blood mononuclear cells, and decreased concentration of Plasma acute-phase protein.^[35]^ Earlier research on same composition (BCM-95) when combined with *Boswellia serrata* reduced pain-related symptoms in patients with OA and shown to be superior to those of celecoxib (NSAID) for treating knee OA.^[36,37]^ Curcuminoid -essential oil of turmeric blend showed significantly better results in active rheumatoid arthritis on comparison with diclofenac sodium.^[38]^

The favorable efficacy of combination therapy was observed due to analgesic/anti-inflammatory properties of curcumin that has been attributed to its ability to inhibit COX-2, which results in the suppression of prostaglandin synthesis. Further, curcumin has been shown to suppress several pro-inflammatory cytokines and mediators of their release such as tumor necrosis factor-alpha, interleukin (IL)-1, IL-8 and nitric oxides synthase.^[39]^

In the study, it is found that less number of patients required additional rescue analgesics while receiving combination of curcuminoid complex and diclofenac compared to diclofenac monotherapy depicting more stable pain control with combination therapy. Moreover, significantly less number of patients in curcuminoid complex plus diclofenac group reported AE compared with diclofenac monotherapy. This favorable efficacy and safety profile in curcuminoid complex plus diclofenac may be due to concurrent use of drugs with different mechanisms of action or pharmacokinetics may be more effective and less toxic than each of the mono-therapeutic regimens alone. The rationale for combining curcumin and NSAID from the fact that both drugs inhibit COX-2 by different mechanisms - curcumin down-regulates COX-2 mRNA and protein levels, while NSAID inhibits COX enzyme activity directly by binding to its active site.^[14]^ Anti-ulcer effect of curcumin is in consistent with the previous reports that suggested that curcumin acts as a potent antiulcer compound, protecting against gastric mucosal injury, and suppresses the proliferation of *Helicobacter pylori*.^[16]^ The mechanism of antiulcer activity of curcumin has also been understood. The local inflammatory cytokine IL-6 primarily activates neutrophils, lymphocytes and monocytes/ macrophages at the inflammatory site in stomach, which in turn initiates different oxidative bursts toxic metabolites and lysosomal enzymes responsible for local tissue damage in peptic ulcer disease. Thus, the proinflammatory IL-6 could predict more precisely the severity and duration of inflammation, particularly in its acute phase, than tumor necrosis factor-alpha. Curcumin exerts its anti-ulcer activity not only by affecting oxidative stress and total antioxidant capacity but also by inhibiting IL-6 secretion and preventing apoptosis in a dose dependent manner.^[40]^ According to another published study, curcumin protects gastric damage by efficient removal of H_2_O_2_ and H_2_O_2_ -derived SOH by preventing peroxidase inactivation by NSAID.^[18]^ Further, there is also an evidence for possible involvement of glutathione in the curcumin-mediated gastroprotection.^[17]^ The addition of curcuminoid complex to NSAIDs may potentiate the analgesic effect and due its gastroprotective effect along with its antiulcer effect may help to reduce the GI side effect of NSAIDs.

In clinical trials, measure of pain relief is more preferred over pain severity as it is not dependent on initial pain severity, equality of the change in different parts of scale and variation in the patient's expression. The effectiveness of any particular treatment can be accurately measured by the pain relief which is the change between pain score after treatment and initial pain score. As long as all data points are in between the ’no treatment’ and ’perfect analgesic", more steeper line is directly proportional to better treatment efficacy. The effect of the slope variation and its effect on the mis-interpretation of the results is already reported.^[41]^ A significant difference in the initial pain score can misguide the interpretation of the result as the effect of treatment. In our study pain relief for curcuminoid complex plus diclofenac and diclofenac are well below the “perfect analgesic” (slope = 1) and above “no treatment” (slope = 0) graph. The slope variation of regression line between the two groups is due to the treatment effect since the contribution of slope change due to the initial pain score is practically nil as the initial pain scores are not different with statistical significance (*P* = .43).

In estimating pain relief, it may not be appropriate simply to compare only the scores before and after treatment, because the magnitude of this difference is limited by the placement of the initial mark.^[42]^ Quantal method measures pain relief based on proportion of patients achieving defined degree of pain relief. Such a method is not suitable for testing drugs which produce moderate pain relief and a more sensitive method is required. Assessment of pain severity based on the percentage change from the initial level and a minimum cut off of 50% in pain relief greatly improves the sensitivity of pain measurement scales.^[41]^ Every patients in curcuminoid complex plus diclofenac group had more than 50% of reduction in Visual Analog scale score from baseline levels compared to 67 patients in diclofenac group with *P* value .24. The study clearly shows the significant analgesic property of combination of curcuminoid complex and diclofenac in comparison with diclofenac sodium.

Based on overall efficacy and safety results, the patient's and physician's global evaluation of treatments also favored combination therapy of curcuminoid complex and diclofenac than diclofenac monotherapy, which reflects the better acceptability of combination therapy of NSAIDs and curcuminoid complex among patients of OA knee. Our findings suggest that the combination therapy of curcuminoid complex and diclofenac 2 times daily is more effective to diclofenac 2 times daily among patients with OA of knee. Significant reduction was observed in diclofenac induced GI side effects in patients who received diclofenac along with curcuminoid complex as compared to those who received only diclofenac. Addition of curcuminoid complex to diclofenac helps to reduce the GI side effects induced by diclofenac and reduced the requirement of H2 blockers. Also the addition of curcuminoid complex to diclofenac reduces the need of other analgesics. Curcuminoid complex due to its high bioavailability can be suggested to give along with diclofenac to get better results if monotherapy is inadequate among patient with OA of knee.

### 4.1 Limitations of the study

The open-label study design without a placebo-controlled group was one of the limitations of the study. The treatment duration of 28 days may not be sufficient enough to assess long term efficacy and prevention of progression of disease as evidenced by structural damage in OA patients. Hence a long-term study is warranted with curcuminoid complex plus diclofenac in OA patients. Efficacy of study groups in treatment of OA was based on subjective measurement of pain and different stages of knee OA were not taken into consideration.

## 5 Conclusion

Combination of curcuminoid complex and diclofenac showed a significant improvement in pain, symptoms, and quality of life when compared to diclofenac, indicates synergistic effect between curcuminoid complex and diclofenac. Addition of curcuminoid complex to diclofenac helps to reduce the GI side effects induced by diclofenac and the combination is better tolerated, more effective and safer than diclofenac alone in patients with OA of knee. Curcuminoid complex with increased bioavailability (BCM-95) and diclofenac could be a better alternative treatment option in symptomatic management of knee OA due to its better efficacy, and safety profile.

## Acknowledgments

All authors would like to thank the subjects for their participation in this study, Dr. Rakesh Ojha for performing statistical analysis and Arjuna Natural Pvt. Ltd, India, for providing the patented and trademark registered curcuminoid complex (BCM-95) capsules as study samples.

Supplementary link:.

## Author contributions

Dhaneshwar Shep made substantial contributions to conception and design, acquisition of data, and analysis and interpretation of data. Chitra Khanwelkar was involved in drafting the manuscript and revising it critically for important intellectual content and gave final approval of the version to be published. Prakashchandra Gade was involved in drafting the manuscript and revising it critically for important intellectual content. Satyanand Karad agreed to be accountable for all aspects of the work in ensuring that questions related to the accuracy or integrity of any part of the work are appropriately investigated and resolved. All authors read and approved the final manuscript.

## Supplementary Material

Supplemental Digital Content
