# Supplementary material for: Efficacy and safety of combination of curcuminoid complex and diclofenac versus diclofenac in knee osteoarthritis: A randomized trial
Source: Medicine (Baltimore). 2020 Apr 17;99(16):e19723. doi: 10.1097/MD.0000000000019723 (PMC7220260; doi:10.1097/MD.0000000000019723)
Supplement: Supplemental Digital Content [file medi-99-e19723-s001.doc]

**Knee injury and osteoarthritis outcome score (KOOS)**

The Knee Injury and Osteoarthritis Outcome Score (KOOS) is a questionnaire designed to assess patient-relevant outcomes following knee injury. The KOOS is patient-administered and assesses five outcomes: pain; symptoms; function in daily living; function in sport and recreation; and knee-related quality of life. The questionnaire format is user-friendly and is given below.

**KOOS Questionnaire**

**PAIN**

P1. How often does patient experience knee pain?

|  | Never | Monthly | Weekly | Daily | Always |
| --- | --- | --- | --- | --- | --- |
| Baseline Visit 1 |  |  |  |  |  |
| Visit 2 |  |  |  |  |  |
| Visit 3 |  |  |  |  |  |

What amount of knee pain has patient experienced the last week during the following activities?

P2. Twisting/pivoting the knee

|  | None | Mild | Moderate | Severe | Extreme |
| --- | --- | --- | --- | --- | --- |
| Baseline Visit 1 |  |  |  |  |  |
| Visit 2 |  |  |  |  |  |
| Visit 3 |  |  |  |  |  |

P3. Straightening knee fully

|  | None | Mild | Moderate | Severe | Extreme |
| --- | --- | --- | --- | --- | --- |
| Baseline Visit 1 |  |  |  |  |  |
| Visit 2 |  |  |  |  |  |
| Visit 3 |  |  |  |  |  |

P4. Bending knee fully

|  | None | Mild | Moderate | Severe | Extreme |
| --- | --- | --- | --- | --- | --- |
| Baseline Visit 1 |  |  |  |  |  |
| Visit 2 |  |  |  |  |  |
| Visit 3 |  |  |  |  |  |

P5. Walking on flat surface

|  | None | Mild | Moderate | Severe | Extreme |
| --- | --- | --- | --- | --- | --- |
| Baseline Visit 1 |  |  |  |  |  |
| Visit 2 |  |  |  |  |  |
| Visit 3 |  |  |  |  |  |

P6. Going up or down stairs

|  | None | Mild | Moderate | Severe | Extreme |
| --- | --- | --- | --- | --- | --- |
| Baseline Visit 1 |  |  |  |  |  |
| Visit 2 |  |  |  |  |  |
| Visit 3 |  |  |  |  |  |

P7. At night while in bed

|  | None | Mild | Moderate | Severe | Extreme |
| --- | --- | --- | --- | --- | --- |
| Baseline Visit 1 |  |  |  |  |  |
| Visit 2 |  |  |  |  |  |
| Visit 3 |  |  |  |  |  |

P8. Sitting or lying

|  | None | Mild | Moderate | Severe | Extreme |
| --- | --- | --- | --- | --- | --- |
| Baseline Visit 1 |  |  |  |  |  |
| Visit 2 |  |  |  |  |  |
| Visit 3 |  |  |  |  |  |

P9. Standing upright

|  | None | Mild | Moderate | Severe | Extreme |
| --- | --- | --- | --- | --- | --- |
| Baseline Visit 1 |  |  |  |  |  |
| Visit 2 |  |  |  |  |  |
| Visit 3 |  |  |  |  |  |

**SYMPTOMS**

These questions should be answered by ascertaining the patient about his/her knee symptoms during the last week.

S1. Does patient have swelling in his/her knee?

|  | Never | Rarely | Sometimes | Often | Always |
| --- | --- | --- | --- | --- | --- |
| Baseline Visit 1 |  |  |  |  |  |
| Visit 2 |  |  |  |  |  |
| Visit 3 |  |  |  |  |  |

S2. Does patient feel grinding, hear clicking or any other type of noise when his/her knee moves?

|  | Never | Rarely | Sometimes | Often | Always |
| --- | --- | --- | --- | --- | --- |
| Baseline Visit 1 |  |  |  |  |  |
| Visit 2 |  |  |  |  |  |
| Visit 3 |  |  |  |  |  |

S3. Does patient's knee catch or hang up when moving?

|  | Never | Rarely | Sometimes | Often | Always |
| --- | --- | --- | --- | --- | --- |
| Baseline Visit 1 |  |  |  |  |  |
| Visit 2 |  |  |  |  |  |
| Visit 3 |  |  |  |  |  |

S4. Can patient straighten his/her knee fully?

|  | Never | Rarely | Sometimes | Often | Always |
| --- | --- | --- | --- | --- | --- |
| Baseline Visit 1 |  |  |  |  |  |
| Visit 2 |  |  |  |  |  |
| Visit 3 |  |  |  |  |  |

S5. Can patient bend his/her knee fully?

|  | Never | Rarely | Sometimes | Often | Always |
| --- | --- | --- | --- | --- | --- |
| Baseline Visit 1 |  |  |  |  |  |
| Visit 2 |  |  |  |  |  |
| Visit 3 |  |  |  |  |  |

S6. How severe is patient's knee joint stiffness after first wakening in the morning?

|  | None | Mild | Moderate | Severe | Extreme |
| --- | --- | --- | --- | --- | --- |
| Baseline Visit 1 |  |  |  |  |  |
| Visit 2 |  |  |  |  |  |
| Visit 3 |  |  |  |  |  |

S7. How severe is patient's knee stiffness after sitting, lying or resting later in the day?

|  | None | Mild | Moderate | Severe | Extreme |
| --- | --- | --- | --- | --- | --- |
| Baseline Visit 1 |  |  |  |  |  |
| Visit 2 |  |  |  |  |  |
| Visit 3 |  |  |  |  |  |

**FUNCTION IN DAILY LIVING**

The following questions concern patient's physical function. This means patient's ability to move around and to look after himself/herself. For each of the following activities please indicate the degree of difficulty patient have experienced in the last week due to the knee.

A1. Descending stairs

|  | None | Mild | Moderate | Severe | Extreme |
| --- | --- | --- | --- | --- | --- |
| Baseline Visit 1 |  |  |  |  |  |
| Visit 2 |  |  |  |  |  |
| Visit 3 |  |  |  |  |  |

A2. Ascending stairs

|  | None | Mild | Moderate | Severe | Extreme |
| --- | --- | --- | --- | --- | --- |
| Baseline Visit 1 |  |  |  |  |  |
| Visit 2 |  |  |  |  |  |
| Visit 3 |  |  |  |  |  |

A3. Rising from sitting

|  | None | Mild | Moderate | Severe | Extreme |
| --- | --- | --- | --- | --- | --- |
| Baseline Visit 1 |  |  |  |  |  |
| Visit 2 |  |  |  |  |  |
| Visit 3 |  |  |  |  |  |

A4. Standing

|  | None | Mild | Moderate | Severe | Extreme |
| --- | --- | --- | --- | --- | --- |
| Baseline Visit 1 |  |  |  |  |  |
| Visit 2 |  |  |  |  |  |
| Visit 3 |  |  |  |  |  |

A5. Bending to floor/pick up an object

|  | None | Mild | Moderate | Severe | Extreme |
| --- | --- | --- | --- | --- | --- |
| Baseline Visit 1 |  |  |  |  |  |
| Visit 2 |  |  |  |  |  |
| Visit 3 |  |  |  |  |  |

A6. Walking on flat surface

|  | None | Mild | Moderate | Severe | Extreme |
| --- | --- | --- | --- | --- | --- |
| Baseline Visit 1 |  |  |  |  |  |
| Visit 2 |  |  |  |  |  |
| Visit 3 |  |  |  |  |  |

A7. Getting in/out of car

|  | None | Mild | Moderate | Severe | Extreme |
| --- | --- | --- | --- | --- | --- |
| Baseline - Visit 1 |  |  |  |  |  |
| Visit 2 |  |  |  |  |  |
| Visit 3 |  |  |  |  |  |

A8. Going shopping

|  | None | Mild | Moderate | Severe | Extreme |
| --- | --- | --- | --- | --- | --- |
| Baseline - Visit 1 |  |  |  |  |  |
| Visit 2 |  |  |  |  |  |
| Visit 3 |  |  |  |  |  |

A9. Putting on socks/stockings

|  | None | Mild | Moderate | Severe | Extreme |
| --- | --- | --- | --- | --- | --- |
| Baseline - Visit 1 |  |  |  |  |  |
| Visit 2 |  |  |  |  |  |
| Visit 3 |  |  |  |  |  |

A10. Rising from bed

|  | None | Mild | Moderate | Severe | Extreme |
| --- | --- | --- | --- | --- | --- |
| Baseline Visit 1 |  |  |  |  |  |
| Visit 2 |  |  |  |  |  |
| Visit 3 |  |  |  |  |  |

A11. Taking off socks/stockings

|  | None | Mild | Moderate | Severe | Extreme |
| --- | --- | --- | --- | --- | --- |
| Baseline Visit 1 |  |  |  |  |  |
| Visit 2 |  |  |  |  |  |
| Visit 3 |  |  |  |  |  |

A12. Lying in bed (turning over, maintaining knee position)

|  | None | Mild | Moderate | Severe | Extreme |
| --- | --- | --- | --- | --- | --- |
| Baseline Visit 1 |  |  |  |  |  |
| Visit 2 |  |  |  |  |  |
| Visit 3 |  |  |  |  |  |

A13. Getting in/out of bath

|  | None | Mild | Moderate | Severe | Extreme |
| --- | --- | --- | --- | --- | --- |
| Baseline Visit 1 |  |  |  |  |  |
| Visit 2 |  |  |  |  |  |
| Visit 3 |  |  |  |  |  |

A14. Sitting

|  | None | Mild | Moderate | Severe | Extreme |
| --- | --- | --- | --- | --- | --- |
| Baseline Visit 1 |  |  |  |  |  |
| Visit 2 |  |  |  |  |  |
| Visit 3 |  |  |  |  |  |

A15. Getting on/off toilet

|  | None | Mild | Moderate | Severe | Extreme |
| --- | --- | --- | --- | --- | --- |
| Baseline Visit 1 |  |  |  |  |  |
| Visit 2 |  |  |  |  |  |
| Visit 3 |  |  |  |  |  |

A16. Heavy domestic duties (moving heavy boxes, scrubbing floors, etc)

|  | None | Mild | Moderate | Severe | Extreme |
| --- | --- | --- | --- | --- | --- |
| Baseline Visit 1 |  |  |  |  |  |
| Visit 2 |  |  |  |  |  |
| Visit 3 |  |  |  |  |  |

A17. Light domestic duties (cooking, dusting, etc)

|  | None | Mild | Moderate | Severe | Extreme |
| --- | --- | --- | --- | --- | --- |
| Baseline Visit 1 |  |  |  |  |  |
| Visit 2 |  |  |  |  |  |
| Visit 3 |  |  |  |  |  |

**FUNCTION IN SPORTS AND RECREATIONAL ACTIVITIES**

The following questions concern patient's physical function when being active on a higher level. The questions should be answered by ascertaining the patient that what degree of difficulty patient has experienced during the last week due to the knee.

SP1. Squatting

|  | None | Mild | Moderate | Severe | Extreme |
| --- | --- | --- | --- | --- | --- |
| Baseline Visit 1 |  |  |  |  |  |
| Visit 2 |  |  |  |  |  |
| Visit 3 |  |  |  |  |  |

SP2. Running

|  | None | Mild | Moderate | Severe | Extreme |
| --- | --- | --- | --- | --- | --- |
| Baseline Visit 1 |  |  |  |  |  |
| Visit 2 |  |  |  |  |  |
| Visit 3 |  |  |  |  |  |

SP3. Jumping

|  | None | Mild | Moderate | Severe | Extreme |
| --- | --- | --- | --- | --- | --- |
| Baseline Visit 1 |  |  |  |  |  |
| Visit 2 |  |  |  |  |  |
| Visit 3 |  |  |  |  |  |

SP4. Twisting/pivoting on the knee

|  | None | Mild | Moderate | Severe | Extreme |
| --- | --- | --- | --- | --- | --- |
| Baseline Visit 1 |  |  |  |  |  |
| Visit 2 |  |  |  |  |  |
| Visit 3 |  |  |  |  |  |

SP5. Kneeling

|  | None | Mild | Moderate | Severe | Extreme |
| --- | --- | --- | --- | --- | --- |
| Baseline Visit 1 |  |  |  |  |  |
| Visit 2 |  |  |  |  |  |
| Visit 3 |  |  |  |  |  |

**QUALITY OF LIFE**

Q1. How often is patient aware of his/her knee problem?

|  | Never | Monthly | Weekly | Daily | Constantly |
| --- | --- | --- | --- | --- | --- |
| Baseline Visit 1 |  |  |  |  |  |
| Visit 2 |  |  |  |  |  |
| Visit 3 |  |  |  |  |  |

Q2. Has patient modified his/her life style to avoid potentially damaging activities to the knee?

|  | Not at all | Mildly | Moderately | Severely | Totally |
| --- | --- | --- | --- | --- | --- |
| Baseline Visit 1 |  |  |  |  |  |
| Visit 2 |  |  |  |  |  |
| Visit 3 |  |  |  |  |  |

Q3. How much is patient troubled with lack of confidence in his/her knee?

|  | Not at all | Mildly | Moderately | Severely | Totally |
| --- | --- | --- | --- | --- | --- |
| Baseline Visit 1 |  |  |  |  |  |
| Visit 2 |  |  |  |  |  |
| Visit 3 |  |  |  |  |  |

Q4. In general, how much difficulty does patient have with his/her knee?

|  | None | Mild | Moderate | Severe | Extreme |
| --- | --- | --- | --- | --- | --- |
| Baseline Visit 1 |  |  |  |  |  |
| Visit 2 |  |  |  |  |  |
| Visit 3 |  |  |  |  |  |

**Scoring Instructions**
The KOOS's five subscales are scored separately: Pain (nine items); Symptoms (seven
items); Function in daily living (17 items); Function in Sport and Recreation (five items) and Quality of Life (four items). A Likert scale is used and all items have five possible answer options scored from 0 (None) to 4 (Extreme) and each of the five scores is calculated as the sum of the items included.

**KOOS Score Calculation**

Scores are transformed to a 0–100 scale, with zero representing extreme knee problems and 100 representing no knee problems.

Formula for transformation of raw scores of each subscale to a 0-100 scale is mentioned below

Transformed scale = 100 – [(Actual raw score x 100) / Possible raw score range]

where raw score and raw score range is mentioned below.

| Scale | Actual raw score = Sum of values of the following items | Possible raw score range |
| --- | --- | --- |
| Pain | P1 – P9 | 36 |
| Symptoms | S1 – S7 | 28 |
| Function in daily living | A1 – A17 | 68 |
| Function in Sports and recreation | SP1 – SP5 | 20 |
| Quality of life | Q1- Q4 | 16 |
